# Dabigatran Etexilate Related Unilateral Adrenal Hemorrhage

**DOI:** 10.7759/cureus.55254

**Published:** 2024-02-29

**Authors:** Turgay Kalinov, Aleksandar Zlatarov, Nikola Kolev, Krasimir D Ivanov

**Affiliations:** 1 Department of General and Operative Surgery, Faculty of Medicine, Medical University of Varna, Varna, BGR; 2 Department of General and Operative Surgery, Medical University "Prof. Dr. Paraskev Stoyanov", Varna, BGR; 3 Department of General and Operative Surgery, University Hospital St. Marina, Varna, BGR; 4 Department of General Surgery, Medical University of Varna, Varna, BGR

**Keywords:** direct oral anticoagulant therapy, unilateral adrenal haemorrhage, adrenal gland neoplasms, adrenal glands, left adrenal gland

## Abstract

A 63-year-old male presented to our clinic with computed tomography data of a large tumor of the left adrenal gland. The formation is highly suspicious for malignancy with central necrosis and hemorrhage, and a total size of 197/183/201 mm. Due to elevated D-dimer values of 7.17 mg/l (reference range <0.5 mg/l), treatment with dabigatran etexilate 2x150 mg was prescribed following a cardiology consult. On the third day of therapy, the patient noticed a large swelling in the left abdominal flank, which caused discomfort. No additional symptoms were reported. No previous abdominal surgical interventions or trauma were reported. Following a thorough physical examination, the patient was referred for a computer tomography that reported a diagnosis of a tumor of the left adrenal gland. Due to the size of the neoplasm, the suspicion of malignancy, compression of adjacent structures, and significant anemia with an Hb of 112 g/L, operative treatment was chosen as the best treatment modality. The mass was reported as a large organizing adrenal hematoma with no suspicion of malignancy on histology. Following a review of available literature, no other cases of unilateral adrenal hematoma with a size of 201x197 mm, following oral anticoagulant therapy with dabigatran etexilate, without any prior surgery or trauma have been reported. Most clinical cases report bilateral adrenal hemorrhage during the postoperative period, following prophylaxis with heparin and the development of heparin-induced thrombocytopenia. The patient underwent operative treatment, after which the patient recovered normally and was discharged from the clinic without complications.

## Introduction

Dabigatran etexilate related unilateral adrenal hemorrhage is a rarely occurring, albeit potentially lethal condition. It is reported in about 1% of autopsies and has a mortality rate of 15% [1]. The leading etiologies are coagulopathies, internal abdominal trauma, pregnancy, stress, anticoagulant treatment, meningococcal sepsis (Waterhouse-Friderichsen syndrome), autoimmune phospholipid syndrome, and others [2]. In some instances, hemorrhage may occur if the adrenal gland is affected by a neoplastic process, for example, pheochromocytoma, or by metastatic disease (e.g., lung cancer). If bleeding disrupts a significant percentage of the adrenal parenchyma, the condition can lead to adrenal insufficiency, which may manifest clinically with hemodynamic instability. Blood count and blood chemistry changes, such as anemia, hyperkalemia, hyponatremia, and others, can be observed. Monitoring hemoglobin levels is essential for therapeutic management in such patients.

The diagnosis of unilateral adrenal hemorrhage is often made difficult due to the nonspecific presentation of the disease and patient complaints, including epigastric pain, pressure in the lumbar area, vomiting, and hemorrhagic shock. Therefore, thoroughly establishing the medical history is vital for therapeutic management. Mandatory components of said history should be any comorbidities, family health history, and oral medication use. The patient should be referred for abdominal sonography and computed tomography with contrast (CT), the gold standard for adrenal mass diagnosis. CT permits the analysis of tumor size, parenchymal engagement, local invasion, and regional lymphadenopathy.

Anticoagulant therapy is increasingly prominent in the treatment of cardiovascular disorders, pulmonary embolism, deep vein thrombosis, and others [3]. Many direct anticoagulants, such as dabigatran etexilate (Pradaxa®), the first warfarin alternative approved by the Food and Drug Administration (FDA) in 2010 [4], have been introduced in contemporary pharmacological treatment. Dabigatran etexilate is a direct thrombin inhibitor, which reduces thrombin-mediated activation of coagulation factors. However, alongside the beneficial effects of anticoagulant therapy, an incorrect dosage may induce adverse drug reactions such as gastrointestinal and parenchymal hemorrhage (with adrenal hemorrhage as a subtype).

## Case presentation

**Figure 1:**
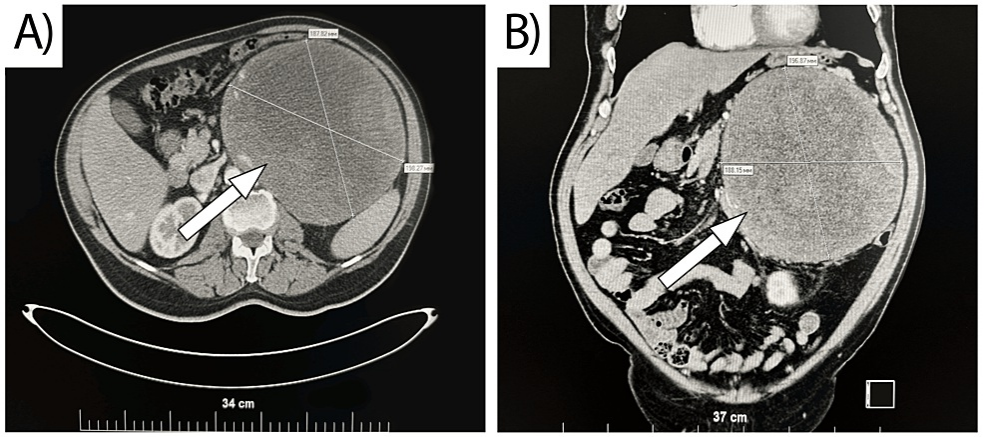
CT of the abdomen with intravenous contrast A) axial plane; B) coronary plane

**Figure 2:**
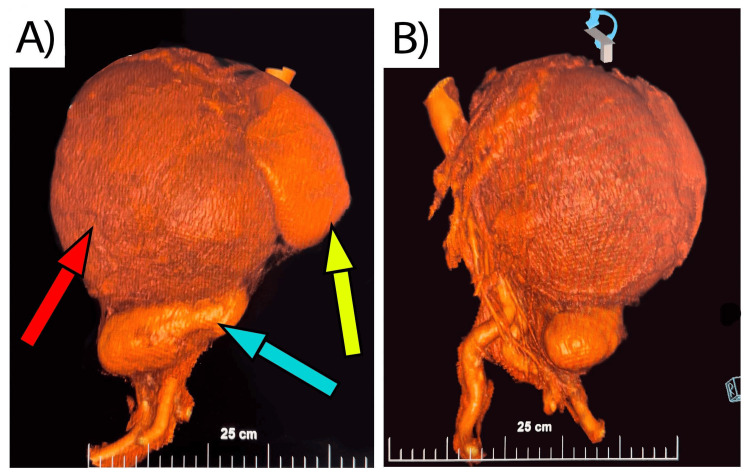
3D reconstruction of the computer tomography of the left kidney, adrenal gland, and hematoma A) Frontal side of view: - red arrow: hematoma - blue arrow: left kidney - yellow arrow: spleen B) Right side of view

A 63-year-old male presented in the First Clinic of Surgery, University Hospital "Saint Marina", Varna, Bulgaria, in October 2022, with a palpable abdominal mass with an adrenal origin confirmed on CT following prophylactic treatment with dabigatran etexilate. The patient’s medical history included well-controlled hypertension, treated with Bisoprolol 5 mg, 1 per evening, and two coronary stents 10 years prior to his initial complaints, which were fever and general malaise a week before admission to the hospital. Due to the symptoms above, a primary care physician ordered a blood chemistry panel, which reported elevated C-reactive protein (CRP) levels of 55.84 mg/l (reference value < 6 mg/l) and elevated D-dimer levels of 7.17 mg/l (reference value < 0.5 mg/l). Despite the negative results of coronavirus disease 2019 (COVID-19) antigen and polymerase chain reaction (PCR) testing, the primary care physician accepted a working diagnosis of severe acute respiratory syndrome coronavirus 2 (SARS-CoV-2). Dabigatran etexilate 2x150 mg was prescribed as anticoagulant therapy after a cardiology consult. Three days following the start of oral anticoagulant therapy, the patient reported significant swelling in his left abdominal flank with concomitant pain and discomfort in the epigastric area. The primary care physician recommended an outpatient abdominal contrast-enhanced CT scan. The radiology report outlined a large left adrenal mass with a size of 197x183x201 mm with zones of necrosis and hemorrhage, suspected to be adrenocortical carcinoma (Figure 1). The volumetric reconstruction revealed compression on the surrounding organs with caudal dislocation of the left kidney, celiac trunk, and upper mesenteric artery, around 5 cm per organ (Figure 2). Regional lymphadenopathy was established as well.

**Figure 3:**
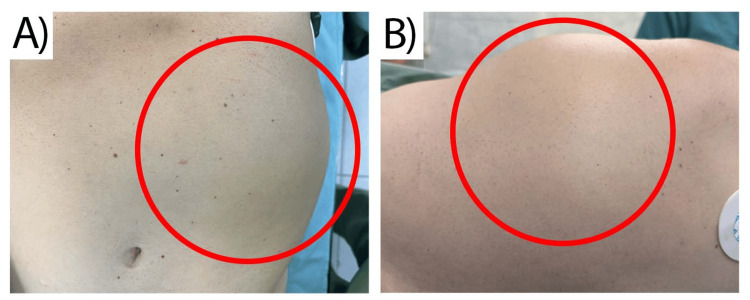
А physical finding in the left abdominal flank A) Frontal side of view B) Left side of view

The patient was admitted to our department, the First Clinic of Surgery, where the physical examination reported an arterial blood pressure of 125/80 mmHg, a body temperature of 36.8 °C, and a pulse of 76 bpm. The integumentary system examination established pale pink skin without decreased turgor, petechiae, or suffusions and no pathological pigmentations. Upon abdominal examination, in the left abdominal flank and left lower rib, we found a large tumor formation with an approximate size of 20 cm (Figure 3). During the palpation, the tumor was relatively immobile, the patient did not report any pain and no changes in color and elasticity were detected on the skin. The inpatient laboratory results are shown in Table 1.

**Table 1:** Laboratory results (L) - decreased indicator; MCV - mean corpuscular volume

Parameters	Result	Reference range (male)
Leukocytes (10*9/l)	8.72	4.0 - 10.0
Hemoglobin (g/l)	112 (L)	130.0 - 180.0
Hematocrit (l/l)	0.364 (L)	0.40 - 0.55
MCV (Fl)	80.4	77 - 100
Platelets (10*9/l)	242	140 - 440
Sodium (mmol/l)	140	132 - 146
Potassium (mmol/l)	4.8	3.50 - 5.50
Chloride (mmol/l)	104	99 - 109
Glucose (mmol/l)	5.3	4.1 - 5.9
Creatinine – serum (umol/l)	71	62 - 115
Urea (mmol/l)	3.5	3.2 - 8.2
Prothrombin activity %	76.0	70 - 130
Prothrombin time sec	13.3	11.5 - 14.8
Prothrombin time INR	1.09	0.9 - 1.15

**Figure 4:**
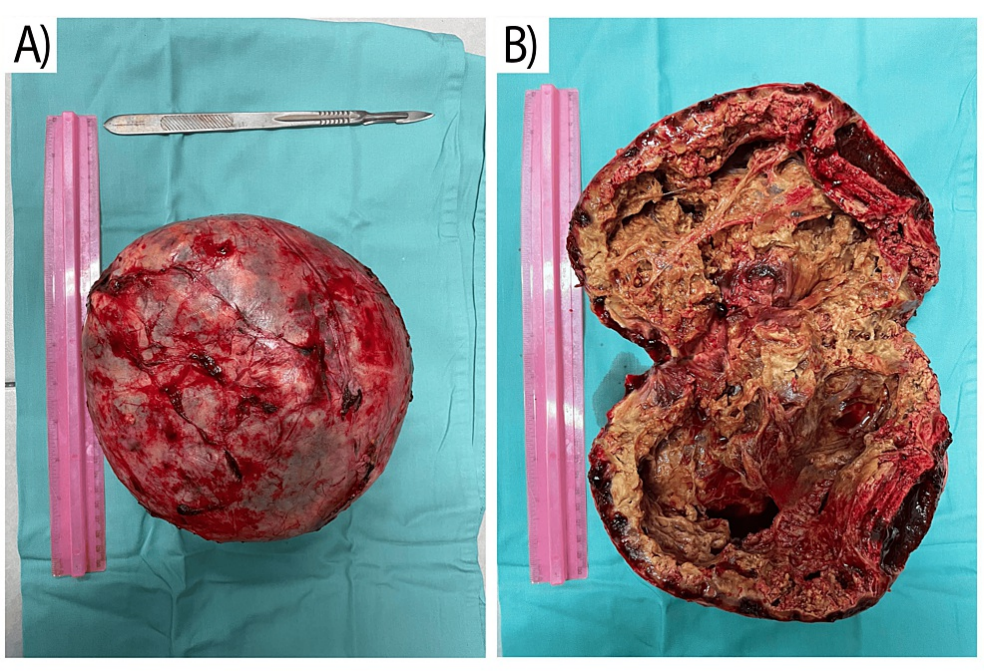
Macroscopic structure of the hematoma and a completely destroyed adrenal gland A) Capsule of the tumor B) Opened tumor

Surgical management for the condition was chosen according to the significant anemia and the findings on CT. Intraoperative antibiotic prophylaxis was carried out with 2x2 grams of cefazolin powder dissolved in 20 ml of physiological solution intravenously, and Metronidazole 2x500 mg/100 ml intravenously. Postoperative antibiotic treatment was not administered. A conventional surgical approach, under general anesthesia, through a standard left subcostal incision, was undertaken due to the size of the tumor formation. The mass was discovered in the retroperitoneal space, with a thick fibrous capsule vascularised by large-caliber venous and arterial vessels. Around 500 ml of cloudy dark liquid was aspirated with an aspiration needle to obtain clear visual access to the left renal hilum and the blood vessels of the adrenal gland; samples were sent for cytological analysis. Cytological examination proved the fluid was benign with characteristics of lysed blood. Following staged surgical mobilization of the adrenal gland, upper, middle, and lower suprarenal arteries, and suprarenal vein were ligated and interrupted sequentially. Hypotonia was not observed after the blood supply interruption. The macroscopic structure of the mass is presented in Figure 4 before and after dissection. It should be noted that intraoperative needle aspiration of the adrenal gland was performed to facilitate mobilization and extraction. An operative wound was closed using standard single absorbable sutures. The patient’s recovery period was unremarkable. The postoperative period continued with 2 liters of infusion solutions of physiological solutions and glucose solution, with intramuscular anesthesia with 4x1 analgin and 2x1/2 lidol. On the first postoperative day, the patient started water intake and movement, and on the second postoperative day, he started on liquid-mushy food. Anticoagulant prophylaxis was carried out with low-molecular-weight heparin Fraxiparin 0.6 until the patient was discharged.

The histological result describes a tumor formation with a weight of 2314 grams, correlating to a hemorrhage in the left adrenal gland with the formation of a giant adrenal hematoma in the early stages of organization (Figure 4).

**Figure 5:**
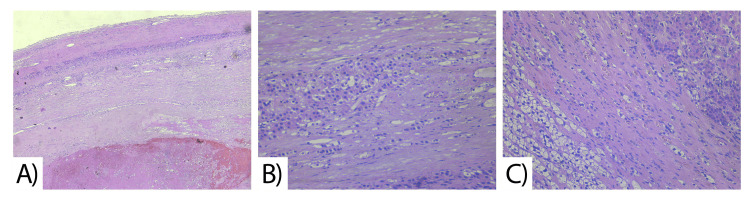
Microscopic structure A) Fibrous capsule and fatty tissue with focal lymphoplasmacytic infiltrate B and C) Amorphous eosinophilic fibrin meshwork, focal erythrocytes, peripheral fibromuscular tissue with areas of enlarged granulation tissue, and atrophic parenchyma of islands of cortical and medullary adrenal tissue

The tumor is clearly limited by a well-formed fibrous capsule, which is characteristic of benign tumors (Figure 5A). The suspected differential diagnosis of carcinoma with necrosis in the adrenal gland was rejected. The histopathological examination did not reveal the presence of cellular and nuclear pleomorphism and necrotic changes, which are characteristic of malignant tumors. Amorphous eosinophilic fibrin meshwork, focal erythrocytes, peripheral fibromuscular tissue with areas of enlarged granulation tissue, and atrophic parenchyma of islands of cortical and medullary adrenal tissue are found. No evidence of invasion of peritumoral tissues and organs was found ( Figure 5B).

## Discussion

We present a clinical case of a large hematoma of the left adrenal gland associated with spontaneous hemorrhage due to oral anticoagulant prophylaxis with dabigatran etexilate treatment. Following an extensive literature review, it was established that this is a novel case of unilateral left adrenal hemorrhage with no associated abdominal surgery or trauma after treatment with this type of anticoagulant, which has not been described until now. The clinical case is also unique due to the hematoma size reaching up to 20 cm in diameter. A clinical case report by Best et al. described a case of a 75-year-old female patient with oral anticoagulant therapy of 150mg of dabigatran etexilate daily following endoprosthetic surgery [5]. On the tenth postoperative day, the patient presents to an emergency department with complaints of shortness of breath, fever, tachycardia, and an arterial blood pressure of 120/57 mmHg. The patient was diagnosed with a massive pulmonary embolism and underwent systemic thrombolysis in an intensive care unit. Following the stabilization of the condition, an abdominal CT scan was performed, establishing bilateral adrenal hemorrhage. Conservative management with fludrocortisone and hydrocortisone yielded beneficial results. Most reported cases describe patients with heparin-induced thrombocytopenia (HIT) following surgical interventions, where there is a bilateral adrenal hemorrhage (BAH).

Unilateral adrenal hemorrhage is a rare condition that can be difficult to diagnose and is established in 0.14-1.1% of autopsied patients [6-8]. The pathogenetic mechanism is not yet fully understood. Ruptures of adrenal medullary vessels, the loose structure of the juxtamedullary complex, and a sudden change in blood pressure may lead to hemorrhage [9,10]. Due to differences in venous drainage, the right adrenal gland is considered more susceptible to hemorrhages. The right adrenal vein drains directly into the lower vena cava (LVC) while the left drains to the left renal or the upper phrenic vein. An increase in venous blood pressure in the LVC may lead to intraglandular hemorrhage. However, the presented case is of a unilateral left adrenal hemorrhage.

A myriad of factors can cause the formation of a non-traumatic adrenal hematoma, including sepsis, pregnancy, and in much rarer cases, oral anticoagulant therapy. Bleeding is the most frequently encountered adverse drug reaction to this class of medicine, and their intake should be strictly monitored. In this case, the anticoagulant therapy was prophylaxis due to a suspicion of a COVID-19 infection and elevated D-dimer levels. The patient did not report any abdominal trauma or surgery, and the only comorbidity was hypertension, which was well-controlled.

The differentiation between bleeding secondary to a neoplasm and spontaneous hemorrhage sometimes poses difficulties. The presence of adrenal hematoma should elicit the differential diagnosis of pheochromocytoma, adenoma, carcinoma, metastatic disease, or myelolipoma [8]. The carcinoma of the left adrenal gland with necrosis and hemorrhage, described in the radiology report of the CT scan, is an indication for emergent surgical intervention. In the available case reports, adrenal hematoma is usually discovered in much earlier stages, and magnetic resonance imaging (MRI) is used to clarify the diagnosis further. A timely diagnosis is vital due to the possibility of developing acute adrenal insufficiency, which may have a lethal outcome.

Management can vary and largely depends on the amount of hemorrhage and the symptoms of adrenal insufficiency. Conservative treatment and monitoring with follow-up imaging should be the initial steps in managing the condition [7,11]. Treatment should be reevaluated if any hemodynamic instability or symptoms of adrenal insufficiency occur. Surgical intervention should be performed if a massive retroperitoneal hematoma is established, as in this case report. Separate from standard surgical treatment, good results have been reported when performing angiography and embolization of adrenal vessels [12,13].

## Conclusions

Unilateral adrenal hemorrhage is a rare condition that can be life-threatening. Our clinical case presented a patient without previous adrenal gland pathology. After taking the oral anticoagulant dabigatran etexilate, in the absence of abdominal trauma, the patient noticed abdominal distension. The performed imaging revealed a suspicion of carcinoma of the left adrenal gland with a central necrosis of 20 cm in size. We performed a left adrenalectomy and after histological analysis, found that it was a hematoma based on a spontaneous adrenal hematoma after taking an anticoagulant. After the operation, the patient recovered successfully, and the histological result proved that it was a hematoma of the left adrenal gland, a consequence of spontaneous hemorrhage from anticoagulant prophylaxis.

If left undiagnosed, it can lead to fatal consequences with the development of adrenal insufficiency, hemodynamic shock, and a lethal outcome. In addition, the nonspecific symptoms may obfuscate the diagnosis. Therefore, a focused and thorough medical history regarding potential trauma, comorbidities, and anticoagulant therapy is vital for the preliminary diagnosis. Medical imaging, such as CT and MRI, have the highest diagnostic value. The treatment in the vast majority of cases can be conservative. However, the presence of a large hematoma, uncontrolled bleeding from the adrenal glands, and any suspicion of an oncological cause or patient status deterioration should be indications for surgical management.
